# Boosting the Photoelectrochemical Performance of Au/ZnO Nanorods by Co-Occurring Gradient Doping and Surface Plasmon Modification

**DOI:** 10.3390/ijms24010443

**Published:** 2022-12-27

**Authors:** Ali Can Güler, Jan Antoš, Milan Masař, Michal Urbánek, Michal Machovský, Ivo Kuřitka

**Affiliations:** Centre of Polymer Systems, Tomas Bata University in Zlin, Tr. T. Bati 5678, 76001 Zlin, Czech Republic

**Keywords:** photoelectrochemical, ZnO nanorods, Au nanoparticles, gradient doping, surface plasmon effect

## Abstract

Band bending modification of metal/semiconductor hybrid nanostructures requires low-cost and effective designs in photoelectrochemical (PEC) water splitting. To this end, it is evinced that gradient doping of Au nanoparticles (NPs) inwards the ZnO nanorods (NRs) through thermal treatment facilitated faster transport of the photo-induced charge carriers. Systematic PEC measurements show that the resulting gradient Au-doped ZnO NRs yielded a photocurrent density of 0.009 mA/cm^2^ at 1.1 V (vs. NHE), which is 2.5-fold and 8-fold improved compared to those of Au-sensitized ZnO and the as-prepared ZnO NRs, respectively. The IPCE and ABPE efficiency tests confirmed the boosted photoresponse of gradient Au-incorporated ZnO NRs, particularly in the visible spectrum due to the synergistic surface plasmonic effect of Au NPs. A gradient Au dopant profile promoted the separation and transfer of the photo-induced charge carriers at the electrolyte interface via more upward band bending according to the elaborated electrochemical impedance spectroscopy and Kelvin probe force microscopy analyses. Therefore, this research presents an economical and facile strategy for preparing gradient plasmonic noble NP-incorporated semiconductor NRs, which have excellent potential in energy conversion and storage technologies.

## 1. Introduction

Photoelectrochemical (PEC) water splitting is a progressive strategy for generating valuable fuels and solving the global energy demand. Since the pioneering research, the selection of photoanode semiconductor nanomaterials has undergone significant changes to emerge as an efficient PEC system [1]. However, the photoanodes still suffer from poor light absorption capacity, charge carrier separation and transport, carrier extraction for the production of electrochemical species and long-term stability in the electrolyte solution [2]. In the last few decades, various metal oxide semiconductors have been extensively employed as photoanodes in water splitting due to their excellent chemical stability, low-cost, tuneable band gaps and appropriate band edge positions [3]. As a multipurpose n-type semiconductor, zinc oxide (ZnO) stands out with its unique features as follows: light-harvesting capability due to fine morphology, high electron mobility assures rapid carrier transport, a large number of surface trap sites improves charge carrier separation and suitable band structure can provide holes with strong oxidation ability [4].

The nanostructure architecture is particularly vital for PEC water splitting applications. Vertically oriented one-dimensional (1D) ZnO nanostructures such as nanorods (NRs) offer merits over their bulk counterparts because of their large aspect ratio, decoupling direction of light absorption, low recombination rate and shorter lateral diffusion length, inducing faster carrier transport [5,6]. Nevertheless, the formation of 1D ZnO nanostructures alone cannot overcome the limitations such as poor charge separation efficiency and visible light absorption due to the direct wide band gap of 3.2 eV. Thus, a tiny fraction of the total solar radiation (~4%) remains to drive the water-splitting process. In addition to this, a theoretical calculation for ZnO semiconductors based on the AM1.5G solar spectrum estimates the maximum photocurrent density of 0.6 mA/cm^2^ [7].

The functionalization of semiconductors with plasmonic nanoparticles (NPs) can improve some of the main PEC limitations, such as charge carrier diffusion and low light absorption [8]. The most salient material among plasmonics is gold (Au) NPs, as it exhibits strong optical absorbance and scattering properties within the visible range. Electromagnetic radiation with a much larger wavelength than the Au NPs can produce collective oscillations of the free electrons in metal across the NPs [9]. These oscillations are the surface plasmon resonance (SPR), which typically lies between 517 and 575 nm [10]. The SPR phenomenon associated with plasmonic NPs helps in suppression of the charge recombination by acting as electron trapping centres, thereby promoting the efficiency of PEC cell [11]. In a study, Au surface plasmons capping provided a six-fold enhancement in the near band gap emission of ZnO NRs [12].

One of the useful strategies to achieve good carrier separation in a semiconductor photoanode is the band bending (built-in electric field) formation, which naturally occurs at the metal–semiconductor interface [13]. In the PEC water splitting field, a gradient dopant profile was recently reported as a new insight to improve the carrier separation through the dispersion of band bending in a more extensive zone in the nanostructured system so that much more electrons can aggregate on the surface and escape to vacuum level [14,15]. For instance, Yu et al. utilised Al gradient doping to increase the power conversion efficiency of ZnO from 2.11% to 3.43% [16]. Rasouli et al. found that Cu gradient doped ZnO photoanodes showed a more efficient photoelectrochemical response than the homogeneous Cu-doped ZnO [17]. The diffusion of Au atoms into materials by high temperature annealing have been reported previously [18,19]. The activation energy for the diffusion of Au in the outer surface of the TiO_2_ nanotubes at 500 °C was calculated to be 67.2 kJ mol^−1^ [20]. However, the diffusion of Au through ZnO NRs forming a gradient doping structure has yet to be reported for PEC water splitting.

Unlike previous reports on similar topics, this research paper investigates the concurrent effects of Au surface plasmon sensitizing and gradient Au dopant distribution into ZnO crystal for the first time. The aim of the simultaneous combination of these two effects in one material is to enrich the photocurrent efficiency. Au NPs sensitization on hydrothermally grown ZnO NRs was achieved by photoreduction method, and a subsequent heat treatment was applied to construct the gradient Au doping. A comparative study of the PEC results is presented to evaluate the performance of the gradient Au-doped ZnO NR photoanode. Besides an elaborated study using standard electrochemical methods, the problem of metal–semiconductor interfacial characteristics was also examined by Kelvin probe force microscopy.

## 2. Results

The gradient Au NPs dopant profile in ZnO NRs was successfully fabricated by hydrothermal technique followed by photoreduction and annealing methods. For comparison purposes of material characteristics, as well as PEC performances, the as-prepared ZnO NRs and Au coated ZnO NRs were also grown on ITO substrates. Hereafter, the as-prepared ZnO NRs, Au-sensitized ZnO NRs, and the gradient Au-doped ZnO NRs will be denoted as ZnO, Au/ZnO, and grad-Au/ZnO, respectively.

### 2.1. Analysis of the Characterization Results

#### 2.1.1. XRD Analysis

**Figure 1:**
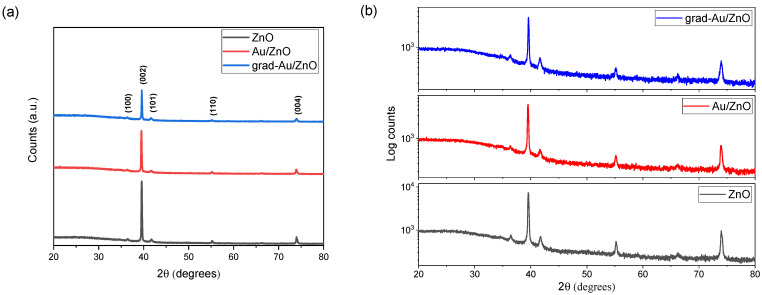
XRD patterns (**a**) in linear and (**b**) in logarithmic scale of ZnO, Au/ZnO, and grad-Au/ZnO NRs photoanodes.

The phase and crystalline properties of the fabricated ZnO, Au/ZnO, and grad-Au/ZnO NRs were examined by XRD measurements. The patterns are shown in Figure 1a in linear, and in Figure 1b at the logarithmic scale to more readily observe small structural changes. The diffraction peak locations of Au/ZnO and grad-Au/ZnO match well with those of ZnO. It is discernible that the sharpest and the most intense diffraction peak appeared around 40° for all the samples is indexed to the (002) plane of hexagonal wurtzite structure of ZnO [21]. The presence of a dominant (002) peak also confirms that the NRs thin films grow along the preferred c-axis [0001], perpendicularly to the ITO substrate. No diffraction peaks of the Au nanocrystal were observed. It is probably due to the low content of the Au NPs (1.7 at.% by EDX). Grad-Au/ZnO showed a significant decrease in the peak intensity of (200). This decrease may indicate structural deterioration on the ground of Au_Zn_ defects or Au segregation at the grain boundaries [22].

The deviation from ideal crystallinity induces peak broadening in the diffraction profile. Several factors that contribute to peak broadening are instrumentation, crystallite size and microstrain. When that crystallite size is more predominant in peak broadening than lattice strain, the diffraction peaks are radically Lorentzian in shape. Hence, a simple expression was used to remove instrument broadening using standard diffraction patterns of LaB_6_:

(1) $$\beta_{observed}=\beta_{specimen}+\beta_{instrument}$$

The average crystallite size (*D*) of the oxides was calculated from Scherer’s equation for (002) of ZnO crystalline domains [23].

(2) $$D=\frac{\left(0.9\lambda \right)}{\beta \cos\theta }\;$$

where *λ*, *β* and *θ* stand for the wavelength of the radiation source, the full-width half maximum (FWHM) and Bragg’s angle, respectively. The calculated crystallite size for the samples is presented in Table 1.

#### 2.1.2. Morphology Analysis by SEM

**Figure 2:**
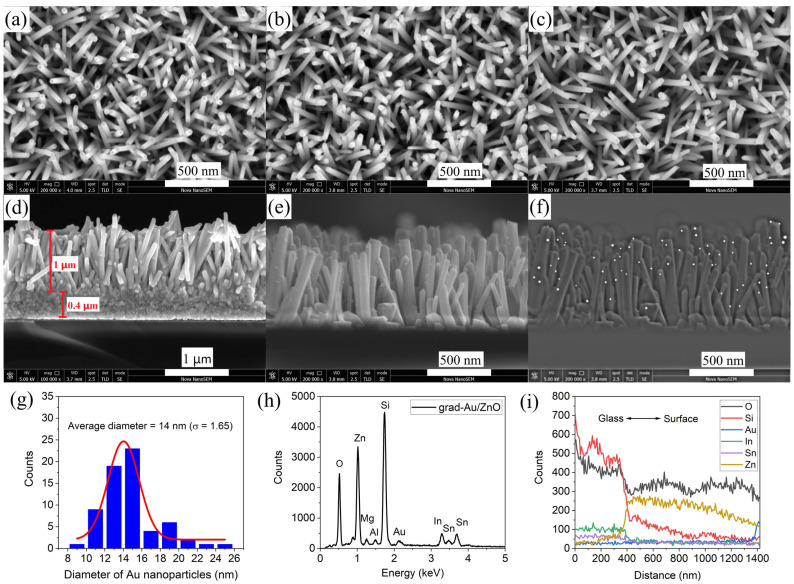
SEM micrographs of NRs (**a**) ZnO, (**b**) Au/ZnO, (**c**) grad-Au/ZnO, cross-sectional views of NRs (**d**) ZnO with the seed layer and (**e**) grad-Au/ZnO, (**f**) corresponding processed image for (**g**) Au NPs size distribution, and (**h**,**i**) EDX spectrum and line profile across grad-Au/ZnO.

The surface morphology and composition of the thin film’s surface was assessed by SEM. The top-view SEM micrographs are indicated in Figure 2a–c. As can be seen, ZnO NRs are vertically grown as a continuous overlay. In Figure 2d, cross-sectional SEM micrograph of ZnO NRs film presents a good quality of ZnO seed layer and also verifies the columnar growth of NRs that is perpendicular to the substrate surface. The measured lengths of ZnO NRs and seed layer from their cross-sectional area are ~1 μm and ~0.4 μm, respectively. The cross-sectional view in Figure 2e is of grad-Au/ZnO exhibiting the sparse but homogeneous Au loading along the ZnO NRs. As indicated in Figure 2f, this micrograph was processed in the Image J software with the help of several plugins to acquire Au NPs diameter distribution on the surface of ZnO NRs in Figure 2g. The average particle diameter of Au was estimated to be 14 nm for 66 particles. The typical SPR effect of Au plasmons requires NPs with sizes varying between 0–100 nm [24]. Thus, gradient doped Au particle size distribution of ZnO photoanode is convenient for SPR enhanced PEC water splitting process.

The elemental composition of samples was identified by the data collected by EDX analysis in Figure 2h. Besides the minor impurities and the intense peak of the Si element detected from the glass substrates, the other intense peaks account for the presence of O, Zn, Sn, and Au constituent elements. Additionally, the line profile scan on the cross-sectional surface of grad-Au/ZnO in Figure 2i evinces that the Au NPs doping concentration gradually increases towards the surface of the ZnO NRs layer.

#### 2.1.3. Morphology Analysis by TEM

**Figure 3:**
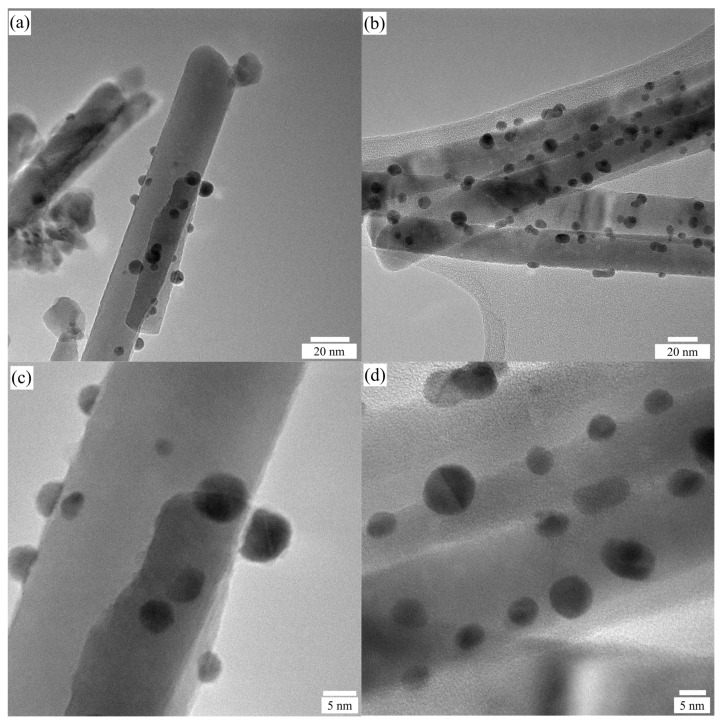
TEM images of (**a**) Au/ZnO and (**b**) grad-Au/ZnO, and HRTEM images of (**c**) Au/ZnO and (**d**) grad-Au/ZnO.

TEM and HRTEM were used to characterize the microstructures of ZnO NRs sensitized with Au NPs, as well as gradient-doped Au NPs. Figure 3a,b reveal low magnifications of TEM images of Au/ZnO and grad-Au/ZnO, respectively. Each Au NPs is almost spherical with dark contrast. The decoration of Au NPs along the crystal facets of ZnO NRs for both samples is very uniform. The Au NPs are distributed in the range of 3–8 nm for Au/ZnO. The annealing of these films at 650 °C results in slightly larger particles with the size distributions of 5–12 nm. Since the amount of photoreduced Au NPs is equal in both samples, there is higher probability for coalescence for Au NPs after annealing. Besides, it is not possible to determine if there are Au NPs on the inner surface of ZnO NRs. Additionally, high-resolution TEM images of Au/ZnO in Figure 3c and grad-Au/ZnO in Figure 3d demonstrate that the Au NPs are firmly adhered to the ZnO surface, leading to well-structured interface between ZnO NRs and Au NPs.

#### 2.1.4. KPFM Analysis

**Figure 4:**
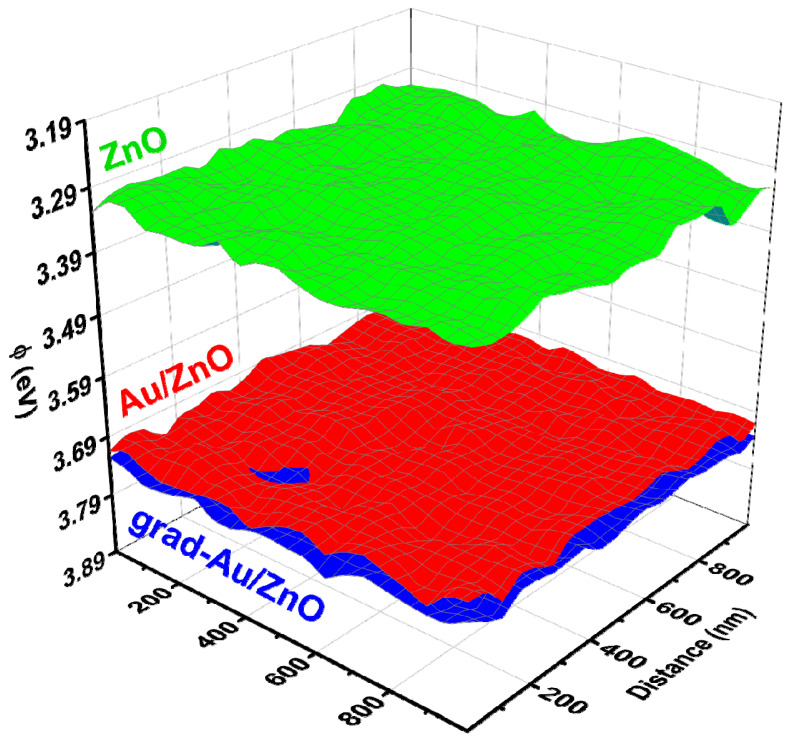
Work function with respect to variations in CPD over photoanode samples.

To verify the effect of gradient Au doping, we conducted Kelvin probe force microscopy (KPFM) to characterize the shift of flat band potential. KPFM accurately exploits long-range Coulomb interactions between conductive tip and sample [25]. This interaction allows the mapping of local electric charge and contact potential distribution. The working principle of KPFM is based on the Fermi alignment of tip and sample; therefore, the local contact potential difference (CPD) depends on the work function difference of these two [26]. Figure 4 reveals work function mapping recorded on 1 μm^2^ of photoanode area. The colour fluctuation across the surfaces indicates the localised variation of the CPD. In addition, the quantitative data was calculated by taking the average of the relative variations in the CPD images. The average CPD value is about 970 mV for ZnO NRs, while it is in the range of 550–580 mV for Au/ZnO and grad-Au/ZnO. After calibration of the tip work function using an HOPG standard sample, the work function (Φ) of the samples was calculated by the following relation.

(3) $$\Phi_{tip}-{\;\Phi }_{sample}=eV_{CPD}\;$$

where *e* is the elementary charge. The resulting work function values of the photoanode samples are shown in Table 2. It is interesting to compare the calculated work function values with previously reported results. A systematic investigation reveals the effect of annealing on the work function values ranging between 2.96–3.46 eV and 4.08–4.22 eV for calcined and uncalcined ZnO nanocrystalline thin films, respectively [27]. These results are in good harmony with the value presented here for ZnO NRs. It is also clearly evident that the presence of Au on ZnO resulted in a work function increment by 0.4 eV. This increase is comparable to what has been measured by KPFM on Au modified ZnO NRs in a study where work function increases due to the increase in electrical resistance or the Schottky barrier formation between Au and ZnO, since it is defined as the energy required to liberate Fermi electrons [28]. The Schottky barrier at a semiconductor/metal interface can exist when the work function of the metal is higher than the electron affinity of the semiconductor. Therefore, a Schottky barrier naturally forms at Au/ZnO and grad-Au/ZnO junctions because the work function of Au (5.1 eV) is larger than the electron affinity of ZnO (4.2 eV), leading to a potential barrier for electrons and to a significant upward bending [29,30]. The work function of grad-Au/ZnO was found to be slightly larger than that of Au/ZnO. A previously reported larger work function obtained after S gradient doping was associated with a larger band bending [31]. Eventually, the presence of the more upward band bending with gradient Au incorporation can block the back transfer of photogenerated charge carriers from the conduction band of ZnO to the electrolyte and improve the photocurrent density.

#### 2.1.5. Optical Property Analysis

**Figure 5:**
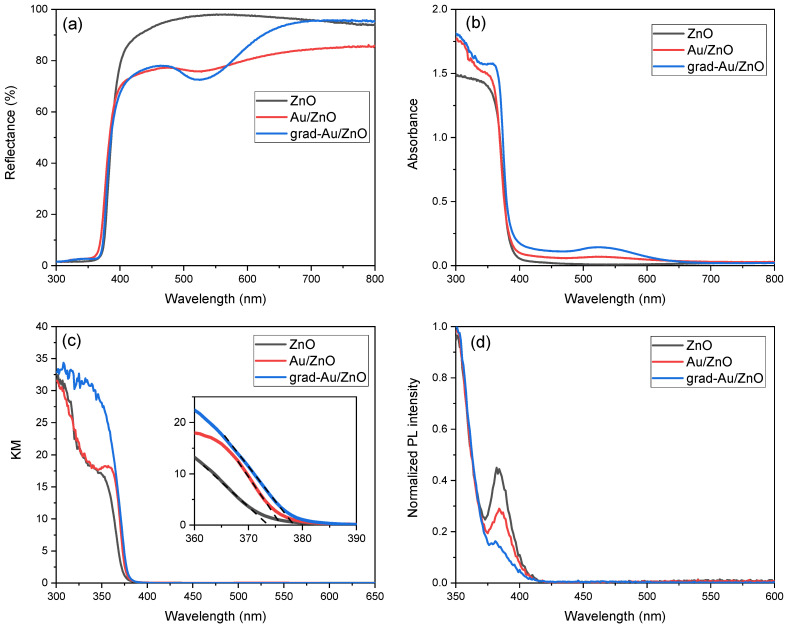
Spectra of (**a**) Reflectance; (**b**) Absorbance; (**c**) Corresponding KM plots; and (**d**) Photoluminescence of ZnO, Au/ZnO, and grad-Au/ZnO NRs.

The spectral properties of nanosized films were studied by the help of DRS. In Figure 5a, the characteristic excitonic absorption peak of ZnO semiconductor appeared around 380 nm for all samples, similarly as in [32]. A reflectance valley around 520 nm was observed for Au/ZnO. As soon as this film is subjected to the heat treatment, the LSPR band becomes more apparent around 525 nm, which is another convincing proof that the gradient Au NPs incorporation contributes the visible light absorption. The absorbance spectra in Figure 5b complementarily displays the slight red-shift of LSPR band in grad-Au/ZnO, indicating the strong interaction between ZnO and Au [33]. The phenomena of diffusion and broadening of the Au atoms into the matrix cause changes in size and distribution of NPs. These are main factors to tune the LSPR band position by uprising the scattering-to-absorption ratio [10]. This modification is supported by the TEM results, manifesting that the heat treatment induced the growth of Au NPs. Similar LSPR response was also detected for Au embedded TiO_2_ nanocomposites [19,20]. Another aspect for the LSPR position is the dielectric constant of the host matrix since the thermal treatment influences that optical response of the nanocomposite films [24]. This possibility can be excluded as the XRD results did not suggest any phase transformation of ZnO NRs by the heat treatment.

Kubelka Munk transformation (KM) was applied to DRS data to remove the extent of light absorption from scattering. Figure 5c denotes KM plots to determine the optical band gaps of the samples. From Table 2, it is seen that Au sensitizing and gradual Au dopants mildly narrowed the band gap energy of ZnO. There can be several reasons for optical band gap reduction. One is possibly strong p-d coupling between O and Au, which escalates the O 2p energy level and narrows the band gap [34]. Another reason may be the presence of shallow acceptor energy levels generated by Au impurities above the valence band. As a result, the energy required for excitonic transition is reduced. It can be concluded that the absorbance of the ZnO photoanode reasonably improved with the contribution of the Au NPs.

Photoluminescence spectroscopy (PL) is essential to understand the recombination nature of the electron–hole pairs in semiconductors. The PL spectra of ZnO NRs were measured at room temperature after Au sensitizing and gradient Au doping. As shown in Figure 5d, the as-prepared ZnO exhibited a strong near band gap emission (NBE) at around 385 nm. It was found that NBE intensity dramatically reduces after Au decoration. The weakest NBE of grad-Au/ZnO was diminished about four-fold relative to the that of as-prepared ZnO NRs owing to a lower recombination of electron–hole pairs from the conduction band.

The presence of metal NPs on the ZnO surface can change the PL behaviour by different mechanisms. According to the radiating plasmon model of Lakowicz, PL enhancement in semiconductor usually occurs on metal NPs with large sizes through the SPR scattering while the quenching occurs on metal NPs of smaller sizes through surface plasmon absorption [35]. Even though, the photoexcited free electrons of Au NPs would transfer to the conduction band of ZnO (lying lower energy state), they can favourably transfer back to Fermi level of Au NPs. These phenomena would cause a quenching of the NBE of ZnO in its PL spectrum [36]. On the other hand, the electrons from the defect level of ZnO can transfer to the surface plasmons of Au because the energy level of defect states and Fermi level of Au are very close to each other [37]. In this way, the high-density electrons would result in an enhancement of NBE of ZnO. A similar observation has been made for the Au NPs sputtered ZnO microdisks by Zhu et al. [38]. However, this is not probable as defect-related peaks in the visible range are not present in our PL spectra. Noteworthy is that the average size of Au NPs in our samples are small (14 nm); thus, absorption dominating over the scattering process leads to a PL quenching. Consequently, the gradient spatial distribution of Au NPs causing a more band bending coupled with its plasmonic effect is very effective for enhancing charge separation in ZnO, which is favourable for the PEC performance of the material.

#### 2.1.6. Photoelectrochemical Performance Test Results

**Figure 6:**
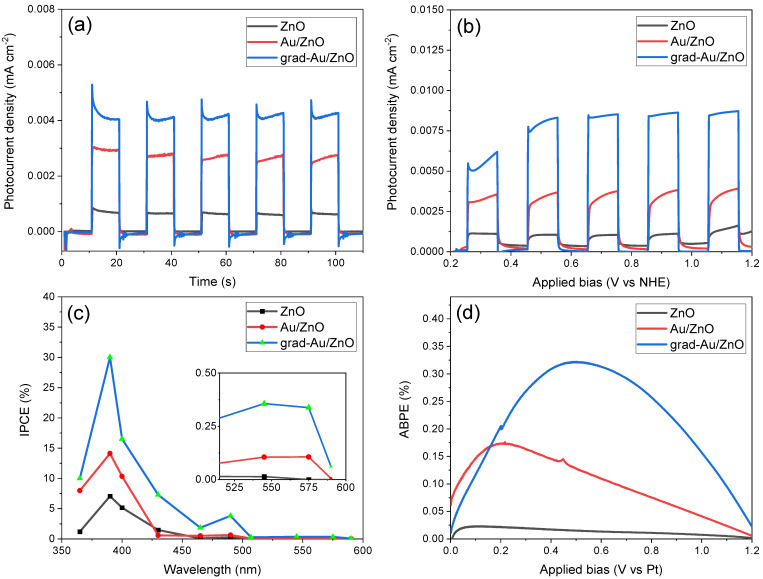
(**a**) Chronoamperometric photoresponse; (**b**) linear sweep voltammetry; (**c**) IPCE at an applied bias of 1.2 V vs. NHE; and (**d**) ABPE efficiencies of ZnO, Au/ZnO, and grad-Au/ZnO photoanodes.

A series of photoelectrochemical measurements were performed to corroborate the effect of Au sensitizing and gradient Au doping on ZnO NRs. The chronoamperometry tests (I-t plots) were carried out under light on-off cycles at an applied bias of 0.2 V vs. NHE (0V vs. Ag/AgCl). Figure 6a represents the transient photocurrent curves in which a similar response was observed for all the photoanodes. Upon illumination at 0.015 mW/cm^2^, the photocurrent escalates to the maximum value and remains almost stable until the light is chopped off, then it dramatically descends to the steady state. Grad-Au/ZnO exhibited the highest photocurrent density value of ~0.0045 mA/cm^2^, which is remarkably higher than Au/ZnO and ZnO. A time-dependent cathodic current was only observed for grad-Au/ZnO when the light is chopped off. This negative current decreases down to zero when the equilibrium at dark is reached. A cathodic current involves the photoreduction of the both surface ${\mathrm{OH}}^{\cdot }$ and chemisorbed H_2_O_2_ species [39]. The transient photoresponse of the samples is also consistent with optical reflectance and photoluminescence spectra, which ensure higher visible light absorption and lower recombination rate for grad-Au/ZnO.

To better probe into photoelectrochemical performance of all prepared photoanodes, LSV voltammograms (I–V plots) were also recorded under light on–off cycles and displayed in Figure 6b. With the increasing applied bias, the I–V response of grad-Au/ZnO NRs substantially increases, while this increase is less significant for other photoanodes. Additionally, the photocurrent density of grad-Au/ZnO was determined to be ~0.009 mA/cm^2^ at an applied bias of 1.1 V (vs. NHE), which is 8-fold and 2.5-fold enhanced compared to the as-prepared ZnO and Au/ZnO NRs, respectively. Au sensitising and gradient element incorporation produced similar photoresponse increase independent of each other in previous studies [40,41,42,43]. We have considered the possibility that the scattering effect of Au NPs with sizes of 14 nm could increase the PEC water splitting activity of grad-Au-ZnO. However, this phenomenon was found to be effective for light absorption for larger Au NPs (55 nm) [33,35]. Therefore, the improved photoanode performance in the case of grad-Au/ZnO can be attributed to the simultaneous impact of SPR effect of Au NPs, which increases visible light absorption capacity, and the increased Schottky barrier height (or band bending), which blocks the back electron transfer, thereby increasing the charge separation efficiency.

IPCE efficiency test was used to determine the specific spectral light response that contributes to the total PEC activity of the prepared photoanodes. The IPCE spectra are demonstrated in Figure 6c. An increased IPCE for grad-Au/ZnO and Au/ZnO can be observed compared with ZnO mainly in the UV region, in agreement with their distinctive photocurrent enhancement. The synergistic SPR effect of Au NPs is again apparent around 550 nm for Au/ZnO and grad-Au/ZnO, where ZnO NRs yields no photocurrent. Au-sensitized ZnO can interact with the incident light in three ways: (1) back reflection by Au NPs, (2) absorbed by the ZnO NRs, and (3) excited SPR of Au [44]. In the first case, the low content of Au NPs decoration (1.7 at.%) is insufficient to scatter the reflected light back to ZnO. In the second case, the increased photoactivity of Au NP-sensitized ZnO in the UV region may be assigned to the passivation of surface trap states [36]. In the last case, surface plasmons of Au can decay via various energy transfer processes such as resonance photon scattering, plasmon resonance energy transfer, and hot electrons generation [45]. As stated in the PL discussion, the resonance photon scattering occurs in large plasmonic nanoparticles (over 50 nm), therefore it can be excluded. The plasmon resonance energy transfer describes electric field amplification that arises from the interaction between the plasmonic NPs and the hosting semiconductor. With wavelength-dependent finite difference time domain (FDTD) method, Bueno-Alejo et al. showed that total enhanced electric field at 365 and 405 nm in ZnO decorated with 15 nm Au NPs was slightly stronger than 460 nm [46]. Considering the consistent sizes of Au NPs, this result can support the fact that improved IPCE of Au-sensitized ZnO photoanodes are due to the increased optical absorption, especially around the UV region. In our opinion, the gradient doping occurring with thermal treatment reinforces the coupling of Au NPs and ZnO NRs, which may also explain stronger field enhancement at the interface. Additionally, absorbed photons can be utilized more efficiently owing to the increased charge carrier density and charge separation by the gradient doping profile. The same result was obtained due to improved charge separation in the gradient P incorporated Fe_2_O_3_ formed by the similar thermal process [41]. Lastly, the hot electrons upon SPR excitation can transfer to ZnO by leaving the energetic holes at Au for water oxidation utilization. Therefore, the enhanced PEC activity in the visible region is primarily ascribed to the hot electron generation.

In addition to that, the ABPE efficiency was used to quantify the photoresponse of the samples as a function of an applied voltage. As shown in Figure 6d, the highest efficiency has been achieved for the grad-Au/ZnO photoanode at an applied bias of 0.5 V vs. Pt electrode. These results confirm that the gradient Au dopant profile can markedly improve the PEC capacity of ZnO NRs photoanode.

#### 2.1.7. EIS Spectroscopy Results

**Figure 7:**
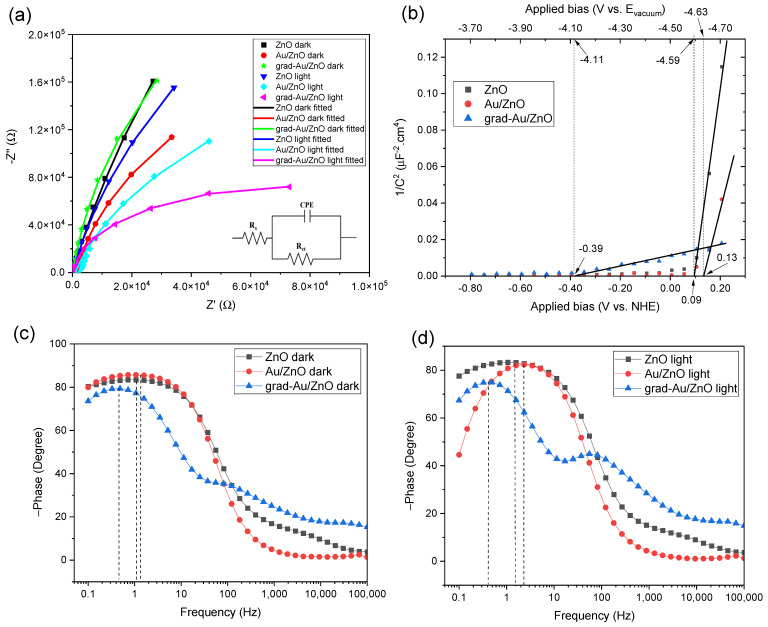
(**a**) EIS data in the Nyquist plots in the absence and presence of illumination (the inset is the equivalent circuit model used for fitting), (**b**) Mott-Schottky plots, (**c**) Bode phase plots at dark, and (**d**) Bode phase plots at light for all the photoanodes.

The electrical properties of the nanostructures play a dominating role in their photoelectrochemical activities. EIS study was carried out to investigate the carrier transport properties at the semiconductor electrolyte interface. Figure 7a shows the EIS results interpreted in Nyquist plots in the absence and presence of the light. The light-sensitive nature of all the photoanode samples is readily apparent from the decreasing arc radius of the impedance curves after illumination. The diameter of the semicircle of the Nyquist plot corresponds to the charge carrier resistance, controlling the electron transfer kinetics at the electrode interface [37]. The scattered dots represent the experimental data, and the solid lines represent the results of fitting these experimental data with the equivalent circuit model (simplified Randle’s cell) shown in the inset.

In the equivalent circuit, *R_s_*, *R_ct_*, and constant phase element (CPE) stand for the resistance of electrolyte solution, the charge transfer resistance, and the capacitance phase element, respectively. We utilised the CPE component, because capacitance formed in EIS measurements does not act ideally due to the non-uniform current distribution arising from the inhomogeneous surface of photoanode thin films [47]. The values of *R_ct_* at dark and light conditions are summarised in Table 2. The comparison of the measured *R_ct_* values of the synthesised photoanodes is also consistent with the order of their PEC efficiencies. Accordingly, grad-Au/ZnO possesses the lowest *R_ct_* value under light, thus facilitating the fastest charge transfer that can be ascribed to efficient separation of photogenerated electron-hole pairs and phenomenological resonant effect under illumination. Based on these results, the reduced *R_ct_* appears to be another attributing factor to improved PEC performance of grad-Au/ZnO NRs.

Mott-Schottky (MS) analyses were implemented to derive essential nanosystem characteristics such as flat band potential (*V_fb_*) and charge carrier density (*N_d_*) of hydrothermally grown ZnO NRs after Au NPs sensitising and Au gradient introduction. The measurements were performed without illumination to exempt the influence of photogenerated charge carriers from these essential properties [23]. The MS equation is as stated below:

(4) $$\frac{1}{C_{s}^{2}}=\left(\frac{2}{e\varepsilon_{r}\varepsilon_{0}N_{d}A^{2}}\right)\left(V_{app}-V_{fb}-\frac{kT}{e}\right)$$

where *C_s_*, *e*, *ε_r_*, *ε_0_*, *N_d_*, *A*, *V_app_*, *V_fb_*, *k,* and *T* are the space charge capacitance, the elementary electric charge, the dielectric constant of ZnO, the vacuum permittivity, the carrier density, the contact area of the electrode, the applied bias vs. NHE, the flat band potential, the Boltzmann constant, and the absolute temperature. In MS plots, as implied in Figure 7b, the positive slope confirms the n-type conducting nature of the semiconductor photoanodes. *N_d_* and *V_fb_* can be extracted from the slope and extrapolating the linear incremental part on the voltage axis of MS plots, respectively. The calculated *V_fb_* and *N_d_* values are listed in Table 2. The *V_fb_* value is the direct indicator of the degree of band bending. Compared to ZnO and Au/ZnO NRs, grad-Au/ZnO has a considerably larger *V_fb_* (closer to the vacuum level), corresponding to a more upward band bending due to the formation of Au gradient dopant profile [48,49]. The *N_d_* value of Au/ZnO was found to be higher compared to the as-prepared ZnO NRs, similar to that of the previous report [50]. A more significant increase in *N_d_* was observed for grad-Au/ZnO. The surface doping of Au NPs may redistribute the carrier density of ZnO, promoting the charge carrier kinetics on the account of extra carriers. The electric field in the band bending region force the photoelectrons to move towards the surface so that much more electrons can arrive and escape to vacuum, which helps in achieving a better photocurrent [51].

The Bode phase plots under dark and light conditions are depicted in Figure 7c,d, respectively. The mid-frequency peak of grad-Au/ZnO shifts to a lower frequency region, corresponding to an increase in electron lifetime. The electron lifetime (τ) can be extracted from $\tau =1/\omega_{min}$, where $\omega_{min}$ is the angular frequency at the mid-frequency peak. This signifies the transient processes occurring at photoanode/electrolyte interface. The calculated τ of the samples under light was derived to be 388 ms, 68 ms, and 106 ms for grad-Au/ZnO, Au/ZnO, and ZnO, respectively. The increase in τ of grad-Au/ZnO supports more effective charge separation by means of a larger band bending, which leads to the substantial improvement in the photocurrent.

As the band edges determine whether an electron transfer is possible between the semiconductor and the electrolyte, the conduction band maxima (*E_CBM_*) and the valence band minima (*E_VBM_*) were also calculated by the following equations:

(5) $$E_{CBM}=E_{F}-kT\ln\left(\frac{N_{D}}{N_{C}}\right)\;\mathrm{and}\;E_{VBM}=E_{CBM}-E_{g}$$

where $E_{F}=-eV_{fb}$ is the Fermi level, and $N_{C}={\left(\frac{2m_{h}^{*}kT}{h^{2}}\right)}^{3/2}$ is the effective density of state in the conduction band, where $m_{h}^{*}$ = 0.24 $m_{0}$. In photoelectrochemistry, the potentials are generally given with respect to the normal hydrogen electrode ($E_{NHE}^{o}\left(H^{+}/H_{2}\right)$ = 0 V), whereas the positions of band edges are related to the absolute vacuum scale (AVS), which is customarily adopted as the standard reference in the field of physics [52]. The NHE is reported to lie at −4.5 eV (at 298.15 K) with respect to the vacuum level on the energy scale [53]. The negative sign here comes from the fact that the energy in the physical scale vs. vacuum level moves towards negative values while the absolute potential moves towards positive values: E_(abs)_/[V] = −E_(AVS)_/[eV] [54]. In order to compare results relative to the NHE, the applied bias converted to the absolute vacuum energy scale is also provided on the top axis of the MS plots.

The protonation and deprotonation of the hydroxylated ZnO surface in contact with aqueous electrolytes lead to positive and negative net charges at the surface, respectively. The potential-determining charges regulates the band edges at the surface. A pH-dependent shift of band edge positions must be considered for a semiconductor like ZnO [52,55]. The pH value at which the number of positively and negatively charged ions at the interface are equal is called the isoelectric point (pH_IEP_) for particulate systems or zero point of charge (pH_ZPC_) for a flat surface. The truly meaningful flat band potential to which the predicted band edges should be compared can only be measured at this point [56]. Therefore, Δ*E*_pH_ factor should be considered as a band shift towards the vacuum level. As described by Stevanovic et al. [57], there is zero net potential across the layer of adsorbed ions rather than the small dipole contribution of the solvent due to the interaction of semiconductor material with water molecules. There has been found that the calculated band edges shift towards the vacuum about ~0.5 eV as a consequence of the dielectric polarisation of the water molecules adjacent to the electrode surface. This fact also introduces a constant contributing term Δ*E_dipole_* and the equation is completed [57].

(6) $$E_{CBs,\;\mathrm{pH}}=E_{CBs}^{0}+\Delta E_{\mathrm{pH}}+\Delta E_{\mathrm{dipole}}$$

(7) $$E_{CBs,\;\mathrm{pH}}=E_{CBs}^{0}+0.059\left({\mathrm{pH}}_{\left(\mathrm{ZPC}\right)}-\;\mathrm{pH}\right)-0.5\;\mathrm{eV}$$

where the $E_{CBs}^{o}$ and Δ*E*_pH_ are the energy of the conduction band edge in the absence of a potential drop (corresponding to pH_IEP_) and the potential change of ~0.059 V predicted by Nernst equation for a pH unit. The same equation holds analogically for the $E_{VBs}^{o}$ of VB.

The isoelectric point of ZnO NPs in the solution was observed to be at pH_(IEP)_ 6.4 in [58] while pH_(ZPC)_ 8.8 is reported in [56]. Moreover, until measured, one cannot judge the ZPC point for such a variable system as ZnO simply. Moreover, the dipole contribution is estimated for water molecules while the ionic strength of the used electrolyte is not taken into account. As aforementioned in the experimental section, all the PEC measurements were performed at the pH value of 7. Thus, it should be noted that the predicted band edge positions of the samples (vs. vacuum level) presented in Table 2 contain the surface shift towards vacuum level due to pH effect and the indispensable dipole contribution. On the other hand, the experimental pH value lies in the interval between reported values for ZPC of ZnO, and the estimate of the flat band potential probably has a low error. At this point, we must conclude, that backward calculation of standard potentials, i.e., positions of CB and VB on physical scale could be erroneous without precise ZPC measurement, while the redox potentials for hydrogen evolution reaction (HER), oxygen evolution reaction (OER), and eventually other reactions in water-based systems are easily accessible for pH 7. For a semiconductor to be used for water splitting, the VBM must be above the hydrogen evolution potential at −4.02 eV in case of photocathode, while the CBM must be below the oxygen evolution potential at −5.25 eV to work as a photoanode (n.b., both at pH 7 on the physical scale) [59,60]. Accordingly, the band edge of the samples lies at appropriate energy levels to drive overall water splitting. Compared to other electrodes, the band energies of grad-Au/ZnO shift upward resulting from the higher Fermi level with increased charge density. The results from the work function measurements in Figure 4 indicated that grad-Au/ZnO and Au/ZnO have greater work function values or require more energy to extract electrons than the blank ZnO. However, this scenario is reversed when the semiconductor electrodes are in contact with electrolyte solution, which can be explained by the very poor charge transfer resistance of ZnO NRs at dark compared to Au/ZnO and grad/Au-ZnO in EIS studies.

#### 2.1.8. Transient Photocurrent Analysis and Photostability Test Results

**Figure 8:**
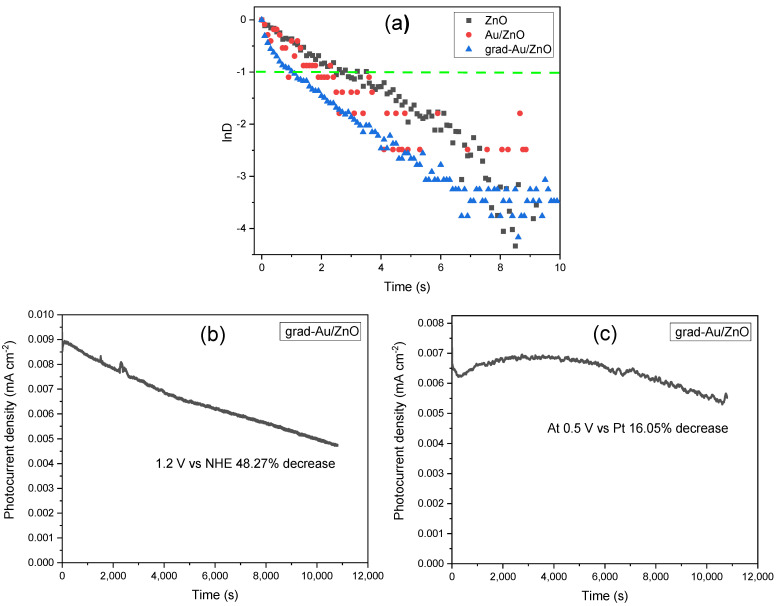
(**a**) Transient decay time curves of all the samples and stability tests for 3 h under illumination (**b**) at an applied bias of 1.2 V vs. NHE in three electrode configuration and (**c**) at an applied bias of 0.5 V vs. Pt in two electrode configurations.

Figure 8a illustrates transient time decay curves (lnD vs. time) to obtain the transient time of the photoanode materials based on the first cycle of the light chopped I-t measurements in Figure 6a. The transient time (τ_D_) can be obtained from $\ln D=-\tau_{D}/t$ and $D=\left(\frac{I_{t}-I_{f}}{I_{i}-I_{f}}\right)\;,$ where *I_t_* is the current at time *t*, *I_t_* and *I_f_* are the initial (t = 0) and final currents (stationary). Assuming mono-exponential decay lnD vs. time, the transient time τ_D_ corresponding to a decrease of *D* to 1/*e* of its original value can be easily read out at lnD=−1. This method is quite rough, but, in many cases, it intuitively shows how long the charge carrier can travel before any recombination occurs [61]. The observed transient time is in the order of: ZnO (*τ_D_* = 2.4s) > Au/ZnO (*τ_D_* = 1.68 s) > grad-Au/ZnO (*τ_D_* = 1.08 s). The larger τ_D_ signifies the suppression of the charge recombination due to the introduced energy barrier at the interface to promote the separation of the injected electrons from the oxidized species in the electrolyte [62]. However, the photocurrent transient decay response of grad-Au/ZnO NRs interestingly did not show linear behaviour, which indicates the decay mechanism is complicated than a single step, involving two or more exponential terms. According to the W. J. Alberly et al., such deviation from the linearity (simple exponential behaviour) is in contradiction with the existence of a surface recombination process, which follows a first-order kinetic in the surface concentration of electrons [63]. As mentioned earlier in chronoamperometric measurements, only grad/Au-ZnO exhibited a cathodic back reaction involving the photoreduction of both the surface OH and chemisorbed H_2_O_2_ species, which are necessary by-products of multi-step OER. A similar diverged decay response was also observed in a study that proposes cathodic back reaction of the electrons with trapped surface holes is responsible for photoanodic one [64]. These authors also concluded that photoanodic transient is due to the build-up holes trapped at photogenerated surface species and concomitant band bending decrease. Furthermore, A. Hagfeldt et al. also suggest that another recombination process escalating the photocurrent decay may be that conduction band electrons begin to reduce photogenerated oxygen species in the electrolyte [65]. As a consequence, observed trends in promoted charge separation in room temperature PL intensity, faster electron kinetics in EIS, as well as higher IPCE and ABPE efficiencies, we conclude that introduction of the gradient structure with its own specific transient time imparts significant mid-products taking part in OER by which the transient response is controlled by the several kinetic rates, helping in promoted PEC activity.

In the current study, the long-term stability of grad-Au/ZnO photoanodes was evaluated using two different approaches aimed at gaining a deeper understanding of the decay mechanism. In the first case, the photoanode was polarized at an applied bias of 1.2 V vs. NHE under continuous illumination over the course of 3 h in the three-electrode system to correlate with IPCE efficiency estimation. This photostability curve of grad-Au/ZnO photoanode is shown in Figure 8b. The photocurrent density is reduced by approximately 48% in 180 min on the occasion of photoanode photo corrosion. Similar, yet drastic photocurrent declination behaviour was reported for ZnO NRs at 1.5 V vs. RHE (~1.2 V vs. NHE) in almost same experimental conditions where the electrolyte is 0.5 M Na_2_SO_4_ with pH 6.8. Herein, the photocurrent decreases to 10% of the initial value only in 30 min and entirely retires after 60 min [66]. In the second approach, the photoanode was polarized at an applied bias of 0.5 V vs. Pt in the two-electrode system to make it parallel with the ABPE efficiency test. In Figure 8c, the photocurrent decrease after 180 min is approximately 16% which is 3 times more stable than the result of the first method. It should be noted that the maximum value of ABPE depends on the open circuit photovoltage (OCPV) or the flat band potential, which is very close to the OCPV. Therefore, this analogy can provide useful information to explore at what potential the photoanode is the most efficient to lose electrons for the water redox potential, which is 0.5 V vs. Pt for grad-Au/ZnO. The photoanode may become more prone to corrosion at the applied potentials greater than this value when exceeding corrosion potential.

## 3. Discussion

**Figure 9:**
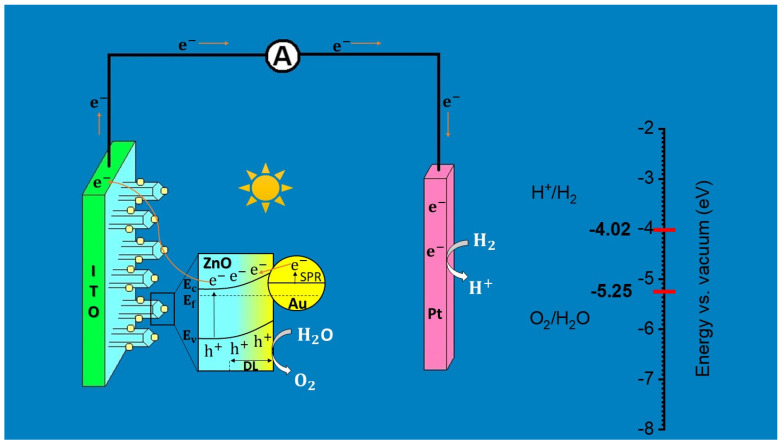
Schematic diagram of photoelectrochemical water splitting process via grad-Au/ZnO.

The schematic diagram of PEC water splitting process by gradient Au-doped ZnO NRs photoanode is presented in Figure 9. In this mechanism, the large depletion region (DL) is generated from gradient distribution of Au dopants, indicated by the yellow gradient colouring. The Au NPs can produce electrons under visible light through SPR process. These photogenerated electrons of Au NPs can be transferred to ZnO conduction band. In fact, the formation of Schottky barrier at Au/ZnO interface, as well as enhanced electric field on space charge region due to more upward band bending, push the current flow of photogenerated electrons towards the electrolyte, enriching the bulk charge separation. However, the extent of electrons vaulting these potential barriers migrate to Pt electrode throughout ITO conductive substrate. In this way, the improved overall PEC activity is completed on both electrodes. Besides, we also showed the HER at −4.02 eV and the OER at −5.25 eV in red on the energy scale in Figure 9.

## 4. Materials and Methods

### 4.1. Synthesis of ZnO NRs

Indium tin oxide coated glass substrates (ITO, 5–15 Ω sq^−1^, Sigma Aldrich, Burlington, NJ, USA) were cleaned by ultrasonication in a mixture of alkaline concentrate (Hellmanex III) and deionized water, isopropanol, and acetone for 10 min, and dried in air atmosphere. Additionally, the UV-ozone cleaning procedure was used for 10 min to remove the remaining contaminants at the surface and improve thin-film adhesion.

A two-step solution growth process was involved in the synthesis of ZnO NRs. The ZnO seed layer was first derived using the sol-gel method in a typical procedure. The precursor solution was prepared by dissolving 0.036 mol zinc acetate dihydrate (Zn(CH_3_CO_2_)_2_.2H_2_O, 99.0%, Penta, Prague, Czech Republic) and 0.036 mol diethanolamine ((CH_2_CH_2_OH)_2_NH, 99.0%, CDH Fine Chemicals, New Delhi, India) in 41.5 mL of isopropanol ((CH_3_)_2_CHOH, 99.7%, Microchem, Ahmedabad, India) at 25 °C.

The resulting mixture was stirred at 50 °C for 1 h and aged for 24 h at room temperature. The sol was then spin-coated onto ITO substrates at 3000 rpm for 30 s. Finally, the wet films were calcined at 400 °C in an air atmosphere for 1 h. Aqueous growth solution (100 mL) including equimolar (0.05 M) zinc nitrate hexahydrate (Zn(NO_3_)_2_·6H_2_O, 98.0%, Sigma Aldrich) and hexamethylenetetramine ((CH_2_)_6_N_4_, 99.6%, Lachner, Neratovice, Czech Republic) and polyethyleneimine (0.50 mL, molecular weight 800, Sigma Aldrich) was preheated for 2 h at 93 °C. Subsequently, the seeded substrates with the conductive faces turned upside down were immersed in this hot solution and kept in a regular oven to grow ZnO NRs at 93 °C for 6 h. The products were ultimately rinsed with deionized water and dried in an oven at 80 °C.

### 4.2. Preparation of Au-Sensitized and Gradient Au-Doped ZnO NRs

**Figure 10:**
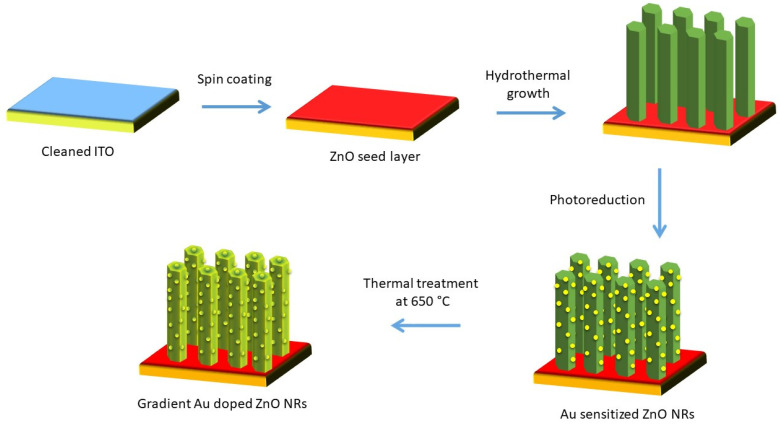
Schematic illustration of fabrication of gradient Au-doped ZnO nanorods.

Figure 10 presents the schematic illustration of the fabrication steps for the gradient Au-doped ZnO photoelectrode. Au NP-sensitized ZnO NRs were produced by a simple photoreduction method. Briefly, aqueous gold metal salt solution (HAuCl_4_.3H_2_O, 10 mM, Sigma Aldrich) was dispersed in deionized water (100 mL). The as-prepared ZnO NRs sample was inserted into this diluted solution irradiated under a UV lamp (8 W) with a wavelength at 365 nm for an hour. The final products were again rinsed with deionized water and dried in an oven at 80 °C. A uniform brownish film formed against the light indicates that gold precursor can be reduced to form Au NPs on the surface of ZnO. To introduce gradient diffusion of Au NPs into the ZnO matrix from top to bottom, these thin films were subsequently calcined at 650 °C in an air atmosphere for 1 h. With this process, the colour of the films turned to speckled purple.

### 4.3. Characterization

The phase identification, the average crystallite size and the unit cell dimensions of the ZnO thin films were analyzed using an X-ray diffractometer (XRD, Miniflex 600, Rigaku, Tokyo, Japan) equipped with Co-K_ɑ_ irradiation source (*λ* = 0.17902 nm), by performing sweeps from 20° to 80° of 2θ angle. The diffractometer was operated at 40 kV, 100 mA, and the step size was 0.04°. The surface morphology and conformation of the ZnO nanostructures were analyzed by scanning electron microscope (SEM, Nova NanoSEM 450, FEI, Hillsboro, OR, USA) with secondary electron and backscatter electron modes. The elemental compositions of the produced metal oxides were determined through energy-dispersive X-ray spectroscopy (EDX) integrated into SEM. Additionally, the cross-sectional line profile EDX images were collected to confirm the successful formation of the gradient doping profile of Au. The microstructure of the prepared nanocomposites was investigated by transmission electron microscopy (TEM) and high-resolution TEM (HRTEM) using a JEM-2100Plus (Jeol, Tokyo, Japan). The surface potential mapping of nanostructures was investigated using Kelvin Probe Force Microscopy (KPFM, Dimension Icon from Bruker, Billerica, MA, USA), a noncontact technique of AFM. The work function of the CoCr tip was calculated by the known work function of highly oriented pyrolytic graphite used as a reference sample. The optical properties of the samples were characterized using diffuse reflectance spectroscopy (DRS) accessory of a UV-Vis-NIR absorption spectrometer Lambda 1050 (Perkin Elmer, Waltham, MA, USA). Further, photoluminescence (PL) spectra of oxide films were examined by the fluorescence spectrometer FLS920 from Edinburgh Instruments equipped with Optistat DN (Oxford Instruments, Abingdon, UK), using diode laser EPLED 330 nm as excitation light source.

### 4.4. PEC and Electrochemical Measurements

The photoelectrochemical tests were performed in three-electrode configuration in a plastic cuvette (3.5 mL) where the samples were used as the working electrode, Ag/AgCl (saturated KCl), as the reference electrode and Pt wire as the counter electrode. The electrolyte was Na_2_SO_4_ aqueous solution (0.5 M) at pH 7. Before all measurements, a nitrogen purge was conducted for 10 min to exclude dissolved oxygen from the electrolyte. The photoanode was in contact with the electrolyte from the side surface of the cuvette in the direction of incident light while its bare area was kept outside of the electrolyte to make contact with electrical connection legs. The samples were irradiated from the front side by the light passed through the electrolyte and cuvette before reaching its surface. The active surface area of the photoanode was estimated to be 0.32 cm^2^. Unless otherwise stated, illumination was provided by The AvaLight-DH-S deuterium halogen source equipped with a fiber optic illuminator with 0.015 mW/cm^2^ light intensity in all PEC measurements. Moreover, the light was chopped electronically by TTL shutter of light source connected to the potentiostat, which ran the photocurrent experiments at given period upon external light stimulus (trigger in). Moreover, the measured electrode potential with respect to Ag/AgCl reference electrode was converted to the normal hydrogen electrode (NHE) scale using E_NHE_ = E_Ag/AgCl_ + 0.2 V.

An electrochemical workstation (SP-200, BioLogic, Orlando, FL, USA) was employed to measure the current-voltage characteristic (I–V) characteristic of the electrodes. The linear sweep voltammetry (LSV) curves were recorded by scanning the potential from 0.2 V to 1.2 V (vs. NHE) with a scan rate of 10 mV/s in 10 s of light on−off cycles. The transient photoresponse of the samples was evaluated by the chronoamperometry measurements under a bias voltage of 0.2 V vs. NHE.

In electrochemical impedance spectroscopy measurements (EIS), an AC signal of 10 mV amplitude was applied to the cell in the frequency range spanned from 100 kHz to 0.1 Hz. The Mott-Schottky plots were collected in the dark at 1 Hz with 20 potential steps.

We have also calculated incident photocurrent conversion efficiencies (IPCE) by $\mathrm{IPCE}=\left(1240J_{ph}\left(\lambda \right)\right)/\left(\lambda P_{light}\;\left(\lambda \right)\right)$, where *J_ph_* is the photocurrent density (mA/cm^2^), *λ* is the incident light wavelength (nm), and *P_light_* (mW/cm^2^) is the intensity of light source at each wavelength. In IPCE measurements, monochromatic LEDs were used as light sources. The applied bias photon-to-current efficiency (ABPE), which is an analogue to the STH (solar-to-hydrogen) efficiency with no bias, was also calculated by $ABPE=\left[J_{ph}\left(1.229-\left|V_{app}\right|\right)\right]\eta_{F}/P_{total}$ in which *V_app_* is the applied bias between the WE and CE in the two-electrode configuration, *J_ph_* is the photocurrent density obtained at *V_app_*, and *P_total_* is the intensity of the light source.

## 5. Conclusions

We presented the facile fabrication of effective ZnO NRs thin films with gradient Au plasmonic NPs incorporation in the radial direction via the hydrothermal technique followed by photoreduction and annealing methods. A promising photocurrent density of 0.009 mA/cm^2^ at 1.1 V (vs. NHE) under 0.015 mW/cm^2^ light intensity was achieved, which is 2.5-fold and 8-fold improved compared to the Au-sensitized ZnO and the as-prepared ZnO NRs, respectively. The EDX line profile analysis verifies that the Au dopant indeed has the gradient concentration profile through radial direction of the ZnO matrix. The optical spectroscopy techniques, IPCE and ABPE efficiency tests, I–V characteristics, as well as the detailed Mott-Schottky and EIS analyses, clearly demonstrate that the enhanced PEC performance can be attributed to co-occurring SPR process and charge separation via formation of more upward band bending of the gradient Au NPs incorporation in the ZnO matrix. The IPCE is three times higher and ABPE is more than ten times higher for ZnO NRs with gradient Au doping in comparison with the as-prepared ZnO NRs. Moreover, we expect that the dual benefit of the plasmonic NPs functionalization, as well as their gradient dopant profile, can be expanded to the fabrication of other plasmonic metal NPs and semiconductor heterojunction structures, which are promising in energy conversion and storage technologies.

## Figures and Tables

**Table 1:** Structural and properties of the samples.

Photoanode	Crystallite Size (nm)	Lattice Parameters (Å)		Cell Volume (Å^3^)
	$d_{hkl}\;(002)$	$\vec{a},\;d_{hkl}(101)$	$\vec{c},\;d_{hkl}(002)$	
ZnO	57.16	3.29	5.29	49.83
Au/ZnO	57.09	3.30	5.28	50.11
grad-Au/ZnO	57.07	3.30	5.28	50.15

**Table 2:** Physical properties: flat band potentials and CBM and VBM positions estimated at pH 7.

Photoanode	Band Gap (eV)	Work Function (eV)	*R_ct_* Dark (kΩ)	*R_ct_* Light (kΩ)	*V_fb_*_, pH = 7_ Dark (V)	*N_D_* (cm^−3^ × 10^21^)	*E_CBM,_*_pH = 7_ (eV)	*E_VBM,_*_pH *=* 7_ (eV)
ZnO	3.32	3.33	3080	1420	−4.63	0.130	−4.37	−7.58
Au/ZnO	3.29	3.72	821	534	−4.59	0.368	−4.35	−7.53
grad-Au/ZnO	3.27	3.74	1430	150	−4.11	5.230	−3.94	−7.09

## Data Availability

Not applicable.
